# Dietary α-Linolenic Acid-Rich Flaxseed Oil Exerts Beneficial Effects on Polycystic Ovary Syndrome Through Sex Steroid Hormones—Microbiota—Inflammation Axis in Rats

**DOI:** 10.3389/fendo.2020.00284

**Published:** 2020-05-27

**Authors:** Ting Wang, Liping Sha, Yiwei Li, Lili Zhu, Zhen Wang, Ke Li, Haixia Lu, Ting Bao, Li Guo, Xiaoxia Zhang, Hao Wang

**Affiliations:** ^1^Department of Pathogenic Biology and Medical Immunology, School of Basic Medical Sciences, Ningxia Medical University, Yinchuan, China; ^2^Endocrinology Department, General Hospital of Ningxia Medical University, Yinchuan, China; ^3^People's Hospital of Ningxia Hui Autonomous Region, Yinchuan, China; ^4^Clinical Medical College, Ningxia Medical University, Yinchuan, China; ^5^Department of Anatomy and Histoembryology, School of Basic Medical Sciences, Ningxia Medical University, Yinchuan, China; ^6^College of Traditional Chinese Medicine, Ningxia Medical University, Yinchuan, China

**Keywords:** FO, PCOS, sex steroid hormones, inflammation, gut microbiota, VMB

## Abstract

Polycystic ovary syndrome (PCOS) represents a common endocrine—metabolic disorder disease with chronic low-grade inflammation and alteration of intestinal flora. Serving as functional food, flaxseed oil (FO), which is rich in plant-derived α-linolenic acid of omega-3 polyunsaturated fatty acids, has been proven to benefit for chronic metabolic diseases. However, the exact role of dietary FO on PCOS remains largely unclear. In the present study, 6-week-old female Sprague–Dawley rats were randomly divided into four groups (eight rats/group), including (a) pair-fed (PF) control (CON) group (PF/CON), (b) FO-fed CON group (FO/CON), (c) PF with letrozole-induced PCOS model (MOD) group (PF/MOD), and (d) FO-fed MOD group (FO/MOD). All rats were fed a standard diet. After 3 weeks of modeling and subsequent 8 weeks of treatment, the rats in diverse groups were euthanized and associated indications were investigated. The results showed that dietary FO ameliorated the disorder of estrous cycle and ovarian morphology. In parallel, dietary FO improved the sex steroid hormone disturbance (luteinizing hormone/follicle-stimulating hormone, estrogen, testosterone, and progesterone), body weights, dyslipidemia, and insulin resistance. Moreover, FO treatment improved plasma and ovary inflammatory interleukin (IL)-1β, IL-6, IL-10, and IL-17A, tumor necrosis factor-α, and monocyte chemoattractant protein-1. Additionally, FO intervention significantly modulated the composition of gut microbiota and vaginal microbiota by increasing the abundances of *Allobaculum, Lactobacillus, Butyrivibrio, Desulfovibrio, Bifidobacterium, Faecalibacterium, Parabacteroides* as well as decreasing the abundances of *Actinobacteria, Bacteroides, Proteobacteria*, and *Streptococcus*, the ratio of *Firmicutes*/*Bacteroidetes*. A decrease in plasma lipopolysaccharide level and an increase in short-chain fatty acids, including acetic acid, propionic acid, butyric acid and pentanoic acid, were determined after dietary FO supplementation. Correlation analysis revealed close relationships among sex steroid hormones, inflammation, and gut/vaginal microbiota. Collectively, this study demonstrated that dietary FO ameliorated PCOS through the sex steroid hormones—microbiota—inflammation axis in rats, which may contribute to the understanding of pathogenesis and potentially serve as an inexpensive intervention in the control of PCOS.

## Introduction

Polycystic ovary syndrome (PCOS) represents a common endocrine—metabolic disorder among women of reproductive ages, with a worldwide prevalence of 4–21% (1). Patients with PCOS largely present metabolic and other clinical features including hyperandrogenemia, hyperinsulinemia, obesity, dyslipidemia, insulin resistance, ovulatory dysfunction, menstrual irregularity, and polycystic ovaries (2–4). Moreover, PCOS may cause an increased risk of metabolic disorders, such as type 2 diabetes mellitus (T2DM), cardiovascular disease, metabolic syndrome, endometrial cancer, infertility, obstetrical complications, psychiatric conditions, and other complications (5–7). Recently, genetic, lifestyle, and environmental triggers have been considered as the main drivers of PCOS (8–11), but the exact pathogenesis remains largely unclear. In view of the limitations of current therapeutics for the metabolic symptoms of PCOS, novel and more practical strategies for the control of PCOS are needed.

Numerous evidences have demonstrated that abnormal sex steroid hormones are crucial parameters in PCOS manifestation and development (12). One of the core pathophysiologic features of PCOS is hyperandrogenism, which is caused by elevated testosterone (T) (13), inhibiting estrogen (E2) production (14, 15). Subsequently, decreased estrogen is associated with increased susceptibility to PCOS (16, 17).

Gut microbiota and vaginal microbiota (VMB) are inextricably linked to the occurrence of PCOS (18–20). Gut dysbiosis in PCOS embodies a decrease in alpha diversity, *Bifidobcterium*, as well as an increase in *Bacteroides* (21, 22). Intriguingly, restoration of gut dysbiosis by dietary prebiotic inulin supplementation contributes to the amelioration of PCOS (23). Regarding VMB, in approximately 70% of healthy women of reproductive ages, predominant *Lactobacillus* exhibits beneficial effects to the vaginal ecosystem *via* generating acidic fermentation products (primarily lactic acid) (24, 25). However, the role of both gut microbiota and VMB in the pathogenesis of PCOS needs to be fully elucidated.

Chronic low-grade inflammation is thought to be a key contributor in the pathogenesis of PCOS, with the main performance of a higher concentration of interleukin (IL)-6 and tumor necrosis factor α (TNF-α) (26). A previous study indicated that toll-like receptor 4 (TLR4), which is closely related with inflammation and immunity, probably contributes to the development of PCOS. Moreover, sterol regulatory element-binding protein 1 may promote the TLR4-induced proinflammatory responses by reprogramming fatty acid metabolism (27).

Accumulating studies have demonstrated that sex steroid hormones were closely associated with dysbacteriosis and inflammation in various metabolic disturbances (12, 28). High-throughput sequencing has shown that sex steroid hormones modulate the microbiota composition of mammals (29). In turn, host hormones can affect bacterial gene expression, bacterial virulence, and growth, with consequences on host physiology (30). In PCOS, dysbacteriosis results in gut mucosal permeability, with a resultant increased translocation of lipopolysaccharide (LPS) from the pathogenic into the systemic circulation (31). Moreover, the translocation of gut-derived LPS to the liver *via* the portal circulation subsequently gets it bound to TLR4 on macrophages (Mψs), which is responsible for driving the production of pro-inflammatory cytokines (32). The amount of gut-derived LPS that translocate to the ovary can subsequently promote the inflammatory cascade, leading to ovarian inflammatory changes (31, 32). In addition to LPS, other metabolites (such as short-chain fatty acids, SCFAs) of the gut microbiota have been demonstrated to regulate gut homeostasis, lipid profile, insulin resistance, and inflammation (33). Thus, chronic inflammation might be reduced by improving the gut/vaginal microbiota dysbiosis in PCOS (21).

Nutritional intervention represents a promising strategy for the treatment of PCOS (34). Omega-3 polyunsaturated fatty acids (PUFAs) were found to have new insights into health profit, such as anti-inflammatory, insulin sensitivity, cellular differentiation, and ovulation (35, 36). Flaxseed oil (FO), which is obtained from the seeds of flax plant, serves as mainly plant-derived omega-3 PUFAs [α-linolenic acid (ALA, 18:3 omega-3)] sourced to non-fish eaters. Dietary FO supplementation possesses the ability to attenuate chronic metabolic diseases (37, 38). Furthermore, FO was previously reported to enhance the insulin level, lower the blood glucose level, and restore the enzymatic antioxidant barrier in diabetic rats by blocking insulin resistance (39, 40). However, the effects and the associated mechanisms of dietary ALA-rich FO intervention on PCOS are largely unknown.

The present study aimed to assess the effects of dietary FO supplementation and mechanisms related to sex steroid hormones, inflammation, and gut microbiota in PCOS rats. Our study may contribute to the integral understanding of the pathogenesis of PCOS and the potential application for the control of the disease.

## Materials and Methods

### Animals and Diet

Six-week-old specific-pathogen-free female Sprague–Dawley (SD) rats (body weight, 193 ± 10 g) were purchased from the Laboratory Animal Center of Ningxia Medical University. All animal experiments were approved by the Ethics Committee of Ningxia Medical University (No. 2016-017). The animals were acclimatized for 1 week in polycarbonate cages at a temperature-controlled room (22 ± 2°C, air humidity 40–70%) under a 12-h light and dark cycle.

All animals were fed with a commercial diet of Keaoxieli Feed Co., Ltd., Beijing, China (46.65% crude protein, 20.73% moisture, 0.09% crude fat, 0.13% crude ash, 0.07% crude fiber, microelement calcium, and phosphorus). FO was purchased from Liupanzhen Square Ecological Agricultural Science and Technology Co., Ltd., Ningxia, China, the fatty acid composition of which was identified by gas chromatography–mass spectrometry (GC-MS) (Table S1). Letrozole was obtained from Hengrui Pharmaceutical Co., Ltd., Jiangsu, China.

### Experimental Design

**Figure 1:**
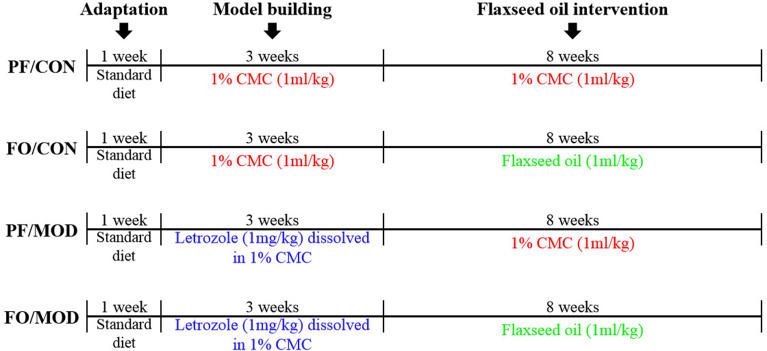
Schematic time diagram of the experimental design.

The schematic time diagram of the experimental design is shown in Figure 1. In brief, after a 1-week period of acclimation with the control diet, 32 female SD rats (6 weeks old) were randomly assigned into four groups (eight rats/group): (a) pair-fed (PF) control (CON) group (PF/CON), (b) FO-fed CON group (FO/CON), (c) PF with letrozole-induced PCOS model (MOD) group (PF/MOD), and (d) PF with FO-fed MOD group (FO/MOD). The MOD groups received letrozole daily for 21 consecutive days at a concentration of 1 mg/kg by gavage, dissolved in 1% aqueous solution of carboxmethlycellulose (CMC) solution, whereas the CON groups were fed daily with 1% CMC, at a concentration of 1 ml/kg, for 21 consecutive days. Vaginal smears were collected daily at 9:00 am and measured with Wright–Giemsa staining for all of the rats. The stage of cyclicity was determined by a microscopic analysis of the predominant cell type. After 21 days of intervention, the rats were under a continuous diestrus phase predominantly exhibiting leukocytes, suggesting that the PCOS model was successful. After modeling, the FO groups received 1 ml/kg of FO daily for 8 weeks by gavage; the PF groups were administrated with 1% CMC (1 ml/kg) daily. The establishment of the PCOS rat model was comparable to that of a previous description (41). Body weight (BW) was measured weekly during 3 weeks of modeling and the subsequent 8 weeks of therapy. The estrous cycle was estimated by collecting vaginal smears daily. At the end of the experiment, all rats were euthanized and associated indications were investigated. Fecal samples and vaginal secretions were snap-frozen and stored at −80°C. The plasma samples were isolated by centrifugation and stored at −80°C.

### Vaginal Smear

Vaginal smears were collected and measured with Wright–Giemsa staining for all of the rats daily at 9:00 am. The stage of cyclicity was determined by a microscopic analysis of the predominant cell type. The four phases of the estrus cycle are comprised of different predominant cells: proestrus, predominantly consisting of nucleated epithelial cells; estrus, predominantly consisting of cornified epithelial cells; metestrus, equal mix of leukocytes, nucleated epithelial cells, and cornified epithelial cells; and diestrus, predominantly consisting of leukocytes. The observation period lasted for 77 consecutive days beginning on the first dose of letrozole until the last day of treatment.

### Ovary Hematoxylin and Eosin Staining

After sacrificing the rats, the ovarian tissue samples were immediately fixed in 4% paraformaldehyde, embedded in paraffin, and sectioned at 4 μm. To evaluate ovary damage, every 10th section (*n* = 8) was mounted on a glass slide, stained with hematoxylin and eosin (H&E), and analyzed using an Olympus light microscope (Melville, NY, USA) by two persons blinded to the origin of the sections. The numbers of cystic follicles and corpora lutea were counted. According to ovarian H&E staining, the number of cystic follicles and corpus luteum as well as the area of three ovarian tissues was detected in diverse groups. Based on the average value of ovarian tissue area in the negative control group, the number of cystic follicles and corpus luteum in the other three groups was corrected. According to the criteria proposed previously, cystic follicles were defined as those follicles devoid of oocytes, displaying a large antrum cavity, an enlarged thecal cell layer, and a thin granulosa cell compartment containing apparently healthy cells (42).

### Determination of Plasma Sex Steroid Hormones

In order to evaluate the level of sex steroid hormones in rats, plasma was obtained to determine the respective follicle-stimulating hormone (FSH), luteinizing hormone (LH), E2, T, progesterone (PROG), as well as sex hormone binding globulin (SHBG), using enzyme-linked immunosorbent assay (ELISA) kits according to the manufacturers' instructions (Shanghai Jianglai Biotech, Shanghai, China). The sensitivities of the assays were 0.1 IU/L, 0.1 mIU/ml, 0.1 pmol/L, 1.0 pg/ml, 0.1 nmol/L, and 0.1 ng/ml for FSH, LH, E2, T, PROG, and SHBG, respectively. For each hormone, the intra- and inter-assay coefficients of variation were <9 and <11%. Each sample was tested in triplicate.

### Plasma Lipid Metabolism

The biochemical indications of lipid metabolism, including plasma total cholesterol (TC), triglyceride (TG), high-density lipoprotein cholesterol (HDL-C), and low-density lipoprotein cholesterol (LDL-C), were respectively determined using AU400 automatic biochemical analyzer (Olympus, Japan).

### Insulin Resistance Tests

Insulin resistance was applied to the homeostasis model assessment of insulin resistance (HOMA-IR), which was calculated as [fasting plasma glucose (FPG) (mmol/L) × fasting insulin concentrations (FINS) (mIU/L)]/22.5. After fasting for 8 h, the FPG from one drop of tail blood was measured by using a standard glucometer (One Touch Profile, Johnson & Johnson, Inc. Milpitas, CA, USA). The plasma FINS was measured with a commercial ELISA kit (Fankewei Biology, Shanghai, China).

### Determination of Plasma and Ovarian Inflammatory Indicators

Plasma and ovarian inflammatory cytokines including IL-1β, IL-6, IL-10, IL-17A, and TNF-α were measured by using ELISA kits according to the manufacturer's instructions (Shanghai Jianglai Biotech, Shanghai, China). The monocyte chemoattractant protein-1 (MCP-1) was measured by using ELISA kits (Fankewei Biology, Shanghai, China). The sensitivities of the assays were 0.1, 1.0, 0.1, 0.1, 1.0, and 0.1 pg/ml for IL-1β, IL-6, IL-10, IL-17A, TNF-α, and MCP-1, respectively. Each sample was tested in triplicate. The plasma LPS levels in each group were examined using a limulus amebocyte lysate kit (Xiamen Bioendo Technology Co., Ltd, Xiamen, China) according to the manufacturer's instruction. Briefly, 50 μl of diluted plasma (1:4 dilutions with endotoxin-free water) was dispensed to each well in a 96-well plate. At the initial time point, 50 μl of the limulus amebocyte lysate reagent was added, respectively. The plate was incubated at 37°C for 30 min. Then, 100 μl of the chromogenic substrate warmed to 37°C was added to each well, and incubation was extended for an additional 6 min at 37°C. The reaction was stopped by adding 100 μl of 25% solution of glacial acetic acid. Optical density at 545 nm was measured with a microplate reader (Thermo Scientific, USA).

### Gut Microbiota and VMB Sequencing Analysis

After 8 weeks of treatment, five rats from each group were randomly selected and placed in sterilized cages. Fresh feces and vaginal secretions of diverse rats were, respectively, collected and immediately stored at −80°C for subsequent DNA extraction. The rats were put into a clean cage with aseptic filter paper, the end of the tail and rectum of which was squeezed gently. Then, feces were collected with sterile tweezers in a sterile Eppendorf tube after defecation and transferred to −80°C for preservation immediately. Moreover, the vaginal orifice of rat was disinfected with alcohol cotton balls. After drying, we took a new aseptic cotton swab and inserted it into the vagina and rotated it gently. Then, the cotton swab with vaginal secretions was taken out and separated by aseptic scissors as well as stored at −80°C immediately for subsequent DNA extraction. The protocols for sample processing, 16S ribosomal DNA gene amplification, sequencing, and statistical analysis of microbiota data were performed as described previously (43).

Extraction of bacterial DNA by cetyltrimethylammonium ammonium bromide (CTAB) was performed by adding the appropriate amount of lysozyme and sample to 1,000 μl CTAB lysate. The mixture was placed in a 65°C water bath and mixed by inversion several times in order to facilitate the complete lysis of the sample. Next, phenol (pH 8.0), chloroform, and isoamyl alcohol were added to the supernatant after centrifugation so that the ratio of the three was 25:24:1, with mixing by inversion and centrifugation at 12,000 × *g* for 10 min. In the same way, chloroform and isoamyl alcohol (24:1) were added to the obtained supernatant, followed by centrifugation. The collected supernatant was added with isopropanol. The mixture was precipitated at −20°C after shaking up and down. Then, the mixture was centrifuged again according to the previous centrifugation conditions. The obtained precipitate was washed twice with 1 ml 75% ethanol. Then, the precipitate was blown dry on a clean bench or air-dried at room temperature. The DNA samples were dissolved in ddH_2_O. If the sample was difficult to dissolve, it needed to be incubated at 55–60°C for 10 min. Finally, 1 μl of RNase A was added to the dissolved DNA sample, which was allowed to be kept at 37°C for 15 min to obtain bacterial DNA. The extracted DNA was stored at −20°C until application.

The DNA sequences involving the V3 and the V4 regions of the 16S rDNA hypervariable regions were amplified by Phusion® High-Fidelity PCR Master Mix with GC Buffer (New England Biolab, USA) using the following primers (5′ to 3′): 341F-CCTAYGGGRBGCASCAG, 806R-GGACTACNNGGGTATCTAAT. The PCR product was analyzed and separated on 2% agarose gel, which was purified using the GeneJE Gel Recovery Kit (Thermo Scientific, USA). The library was constructed using the TruSeq® DNA PCR-Free Sample Preparation Kit in order to carry out Qubit quantitation and library detection. After passing the test, the library was sequenced using the iIllumina HiSeq 2500 platform by Beijing Novogene Technology Co., Ltd., China.

### Measurement of the Feces SCFA Concentrations

Standard acetic acid, propionic acid, butyric acid, and valerate acid at a minimum purity of 98% were obtained from Sigma-Aldrich (St. Louis, MO, USA). Phosphoric acid and ether of analytical grade were purchased from Sinopharm Chemical Reagent Co., Ltd. (Shanghai, China). SCFA (acetic acid, propionic acid, butyric acid, and valeric acid) measurements were carried out on a single quadrupole mass spectrometer (5975B-MSD) equipped with 6890N GC (Agilent Technologies, Santa Clara, CA, USA). The samples were mixed with a QL-866 vortex meter (Haimen, Jiangsu, China) and separated from H1850R refrigerated centrifuge (Xiang Yi, Changsha, Hunan, China). A mixed standard stock solution of the four SCFAs was prepared by dissolving an accurately weighed quantity of ether. Working solution series were prepared by appropriate dilutions of mixed standard stock solutions. A 10-point calibration curve was made by adding the working solutions and an equal volume of IS solution covering a range from 0.05 to 250 μg/ml (0.05, 0.1, 0.5, 1, 5, 10, 25, 50, 100, and 250 μg/ml). All these solutions were stored in a freezer at 0°C prior to use. Fecal samples weighing 100 mg were homogenized in 100 μl of 15% phosphoric acid with 100 μl of 250 μg/ml isohexanoic acid solution as IS and 400 μl ether (70 Hz for 1 min). Subsequently, the samples were centrifuged at 4°C for 10 min (12,000 × *g*), and the supernatants were transferred into a vial prior to the GC-MS analysis. The GC was fitted with a capillary column Agilent HP-INNOWAX (30 m × 0.25 mm i.d. × 0.25 μm) (Agilent Technologies, Santa Clara, CA, USA), and helium was used as the carrier gas at 1 ml/min. Injection was made in split mode at 10:1, with an injection volume of 1 μl and an injector temperature of 250°C. The temperature of the ion source, interface, and quadrupole were 230, 250, and 250°C, respectively. The column temperature was initially 90°C and then increased to 120°C at 10 °C/min, to 150°C at 5°C/min, and finally to 250°C at 25°C/min; and this temperature was kept for 2 min (total run-time of 15 min). The detector was operated in electron impact ionization mode (electron energy 70 eV) using full scan and single ion monitoring (SIM) mode.

### Statistical Analysis

Statistical analysis was performed using GraphPad Prism software 6.01 (GraphPad Software Inc., CA, USA) and SPSS 17.0 (IBM Corp., NY, USA). All experimental data were expressed as mean ± standard deviation of at least three independent experiments. Data were determined by one-way analysis of variance to compare the mean values of variables among the groups. Bonferroni's test or Tukey's *post hoc* test was used to identify the significance of pairwise comparison of mean values among the groups. Moreover, Spearman's correlation analysis was performed to identify the correlations between microbiota and inflammatory indicators. *P* < 0.05 was considered to be statistically significant.

## Result

### Dietary FO Supplementation Improved the Estrous Cycles of Letrozole-Induced PCOS in Rats

**Figure 2:**
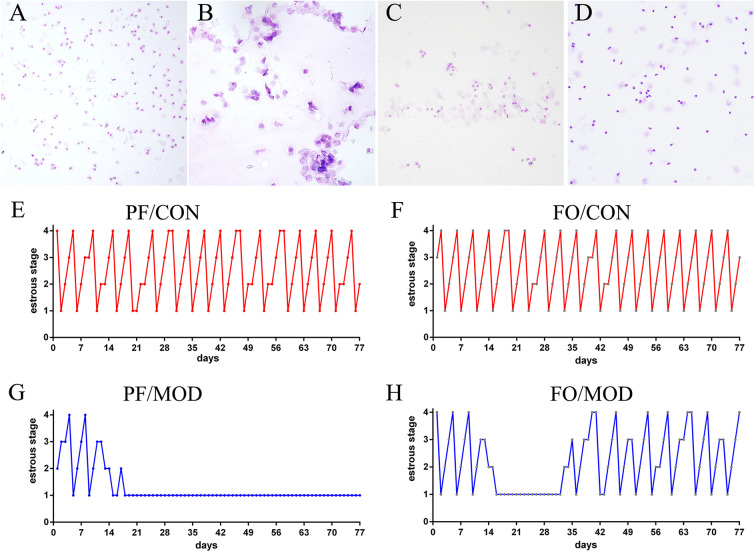
Estrous cycle changes in representative rats from diverse groups. **(A)** Vaginal smears of proestrus stage. **(B)** Vaginal smears of estrus stage. **(C)** Vaginal smears of metestrus stage. **(D)** Vaginal smears of diestrus stage. **(E–H)** Representative estrous cycles of diverse groups: 1, diestrus stage; 2, proestrus stage; 3, estrus stage; 4, metestrus stage. Original magnification (×100).

Wright–Giemsa staining was used to estimate the difference of estrous cycles in diverse groups. Rats in the PF/CON and FO/CON groups exhibited regular estrous cycles of 4–5 days, comprising of proestrus, estrus, metestrus, and diestrus (Figures 2A–F). However, after 18 days of intervention, rats in the PF/MOD group were under a continuous diestrus phase, predominantly exhibiting leukocytes (Figure 2G). Intriguingly, after 20 days of dietary FO intervention, a cyclical variation from nucleated epithelial cells to cornified epithelial cells to a mix of leukocytes, nucleated epithelial cells, and cornified epithelial cells was microscopically observed (Figure 2H), suggesting that dietary FO can improve the disorder of estrous cycle in letrozole-induced PCOS.

### Dietary FO Ameliorated Ovarian Injury in PCOS

**Figure 3:**
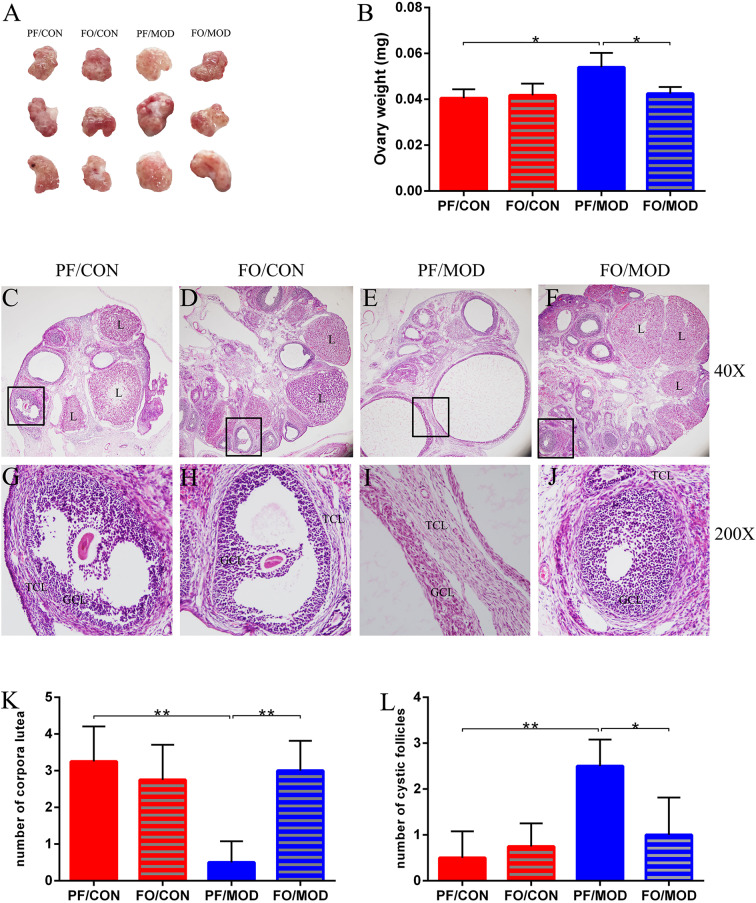
Effects of flaxseed oil on ovarian surface morphologies and weights of diverse groups. **(A)** Ovarian surface morphologies. **(B)** Ovary weights. Effects of flaxseed oil on ovarian tissue morphology of diverse groups with hematoxylin–eosin (H&E) staining. **(C)** PF/CON. **(D)** FO/CON. **(E)** PF/MOD. **(F)** FO/MOD. The larger boxed areas in **(C–F)** (×40) are shown at higher magnification (×200) in **(G–J)**, respectively. **(K)** Numbers of corpora lutea. **(L)** Numbers of cystic follicles. TCL, theca cell layer; GCL, granular cell layer; L, luteum. Original magnification (×40). Data were expressed as mean ± SD. *P < 0.05, **P < 0.01.

Ovary weights in the PF/MOD group were increased with a fairly smooth-white appearance, compared with those in PF/CON group (*P* = 0.0103, Figures 3A,B). However, dietary FO improved the ovarian morphology and the ovary weights (*P* = 0.0153, Figure 3B). Moreover, H&E staining was used to determine the alteration of ovarian pathology in diverse groups (Figures 3C–J). Ovaries in the PF/CON and FO/CON groups possessed follicles and fresh corpora lutea (Figures 3C,D,G,H). The granulosa within follicles in the control group showed multiple layers. Compared to the PF/CON group, the number of cystic follicles in the PF/MOD group was increased (*P* = 0.0027, Figure 3L), but the corpus luteum decreased or disappeared (*P* = 0.0016, Figure 3K), indicating that the ovulatory function of the letrozole-induced PCOS model was impaired. Importantly, dietary FO administration increased the formation of corpora lutea (*P* = 0.0025, Figure 3L) as well as decreased the number of cystic follicles (*P* = 0.024, Figure 3K), demonstrating that dietary FO ameliorated the pathological damage of ovarian tissue and ovulatory function.

### Plasma Levels of Sex Steroid Hormones

**Figure 4:**
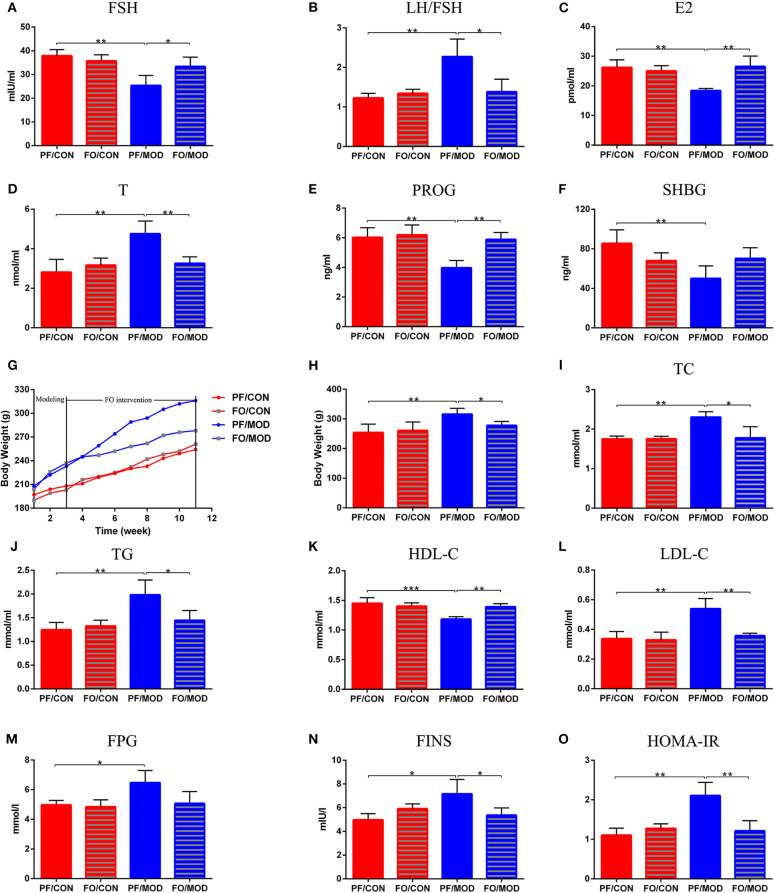
Effects of flaxseed oil on body weights (BWs), lipid metabolism indicators, and sex steroid hormones in diverse groups. **(A)** Body weights growth curve. **(B)** Final BWs. **(C)** Plasma total cholesterol levels. **(D)** Plasma triglycerides levels. **(E)** Plasma high-density lipoprotein levels. **(F)** Plasma low-density lipoprotein levels. **(G)** Plasma follicle-stimulating hormone levels. **(H)** Plasma luteinizing hormone levels. **(I)** Plasma estradiol levels. **(J)** Plasma testosterone levels. **(K)** Plasma progesterone levels. **(L)** Plasma sex hormone-binding globulin levels. Effects of flaxseed oil on insulin resistance of diverse groups. **(M)** Plasma fasting plasma glucose levels. **(N)** Plasma fasting insulin levels. **(O)** Insulin resistance index. Data were expressed as mean ± SD. *P < 0.05, **P < 0.01, ***P < 0.001.

To assess the effects of FO on sex steroid hormones in PCOS, we measured six plasma sex hormones involving FSH, LH, E2, T, PROG, and SHBG using ELISA kit. The levels of LH showed no significant change in the diverse groups (*P* > 0.05, data not shown). However, the levels of LH/FSH and T in the PF/MOD group were significantly higher than those in the PF/CON group (*P* = 0.0038, *P* = 0.0054, Figures 4B,D) or the FO/MOD group (*P* = 0.017, *P* = 0.0063, Figures 4B,D). FSH, E2, and PROG were markedly reduced in the PF/MOD group compared to those of the PF/CON group (*P* = 0.0023, *P* = 0.009, *P* = 0.0024, Figures 4A,C,E) or the FO/MOD group (*P* = 0.0322, *P* = 0.004, *P* = 0.0013, Figures 4A,C,E). In addition, the levels of SHBG in the PF/MOD group were lower than those of the PF/CON group (*P* = 0.009, Figure 4F). The above results demonstrated that dietary FO treatment improved the sex steroid hormones in PCOS.

### Dietary FO Reduced BWs and Dyslipidemia

The body weight growth curve of rats in diverse groups were shown in Figure 4G. There was no significant difference in the initial BWs among the four groups. After 11 weeks of intervention, the BWs in the PF/MOD group were dramatically increased compared to those in the PF/CON group (*P* = 0.0013, Figure 4H). Interestingly, after FO treatment, the final BWs in the FO/MOD group was significantly decreased compared to those in the PF/MOD group (*P* = 0.027, Figure 4H), demonstrating that dietary FO partially reduced the BWs in the PCOS model. To assess the effects of FO on lipid metabolism in PCOS, the plasma levels of TC, TG, HDL-C, and LDL-C were investigated, respectively. As shown in Figures 4I–K, TC, TG, and LDL-C were higher, while HDL-C was lower in the PF/MOD group than those in the PF/CON group (*P* = 0.004, *P* = 0.0052, *P* = 0.0022, *P* = 0.0028, Figures 4I–L) or the FO/MOD group (*P* = 0.0153, *P* = 0.0274, *P* = 0.007, *P* = 0.002, Figures 4I–L). These four lipid indicators showed no significant difference between the PF/CON group and the FO/CON group.

### Dietary FO Reduced Plasma Insulin Resistance in PCOS

To further understand the characteristic insulin resistance in PCOS after dietary FO treatment, we assessed the levels of FPG, FINS, and HOMA-IR in diverse groups. FPG in the PF/MOD group was increased in comparison with that in the PF/CON group (*P* = 0.014, Figure 4M). The FO treatment reduced the FPG levels but without a significant difference (Figure 4M). Intriguingly, FINS and HOMA-IR in the PF/MOD group were significantly higher than those in the PF/CON group (*P* = 0.016, *P* = 0.0018, Figures 4N,O) or the FO/MOD group (*P* = 0.0383, *P* = 0.0055, Figures 4N,O).

### Dietary FO Decreased Plasma and Ovarian Inflammation in PCOS

**Figure 5:**
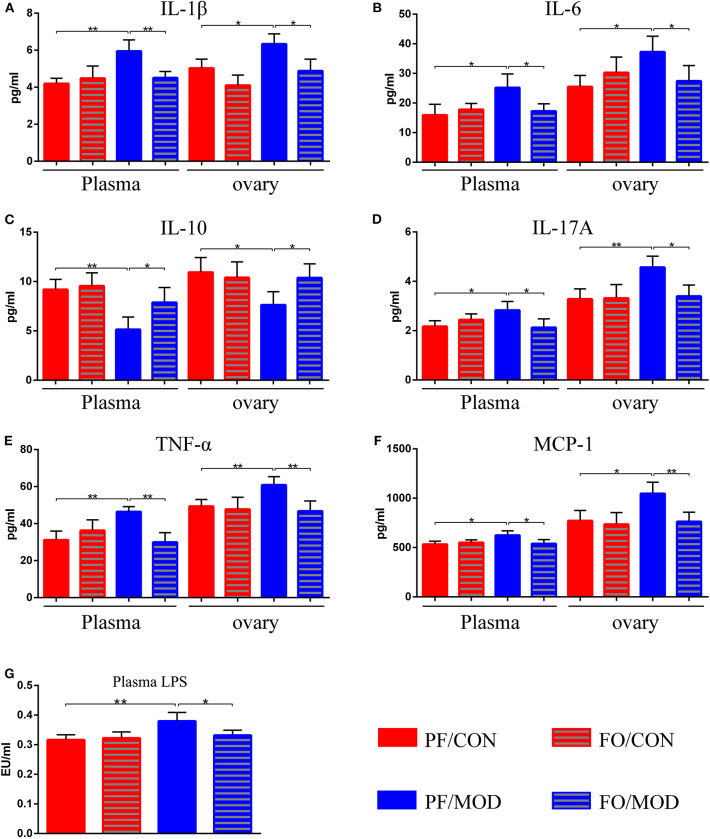
Effects of flaxseed oil on plasma and ovary inflammatory indicators of diverse groups. **(A)** Plasma and ovary interleukin 1β levels. **(B)** Plasma and ovary IL-6 levels. **(C)** Plasma and ovary IL-10 levels. **(D)** Plasma and ovary tumor necrosis factor α levels. **(E)** Plasma and ovary IL-17A levels. **(F)** Plasma and ovary monocyte chemotactic protein 1 levels. **(G)** Plasma lipopolysaccharide levels. Data were expressed as mean ± SD. *P < 0.05, **P < 0.01.

After dietary FO administration, we found elevated plasma levels of IL-1β (*P* = 0.002), IL-6 (*P* = 0.0197), IL-17A (*P* = 0.0218), TNF-α (*P* = 0.0015), and MCP-1 (*P* = 0.0165) in the PF/MOD group in comparison with those of the PF/CON group (Figures 5A,B,D–F). Moreover, dietary FO reduced the plasma levels of IL-1β (*P* = 0.0061), IL-6 (*P* = 0.0231), IL-17A (*P* = 0.031), TNF-α (*P* = 0.0013), and MCP-1 (*P* = 0.0325), respectively, compared to those of the FO/MOD group (Figures 5A,B,D–F). However, plasma IL-10 in PF/MOD was lower than that in the PF/CON group (*P* = 0.0025, Figure 5C) or the FO/MOD group (*P* = 0.0315, Figure 5C). Similar results were observed in the ovarian tissues in the diverse groups (PF/CON vs. PF/MOD: IL-1β, *P* = 0.0121; IL-6, *P* = 0.0112; IL-10, *P* = 0.0165; IL-17A, *P* = 0.0055; TNF-α, *P* = 0.0074; MCP-1, *P* = 0.012. PF/MOD *vs* FO/MOD: IL-1β, *P* = 0.0135; IL-6, *P* = 0.0381; IL-10, *P* = 0.0295; IL-17A, *P* = 0.0104; TNF-α, *P* = 0.0072; MCP-1, *P* = 0.0088, Figures 5A–F). Collectively, dietary FO reduced plasma and ovarian inflammation in PCOS by suppressing pro-inflammatory cytokines and enhancing anti-inflammatory IL-10.

To further assess the effects of FO on gut dysbiosis, permeability, and integrality of gut barrier, plasma-translocated LPS derived from Gram-negative bacteria was detected by the limulus reagent. The plasma LPS concentration in the PF/MOD group was significantly higher than that in the PF/CON group (*P* = 0.0089, Figure 5G). However, the LPS level in the FO/MOD group was decreased compared to that in the PF/MOD group (*P* = 0.0286, Figure 5G), suggesting that supplementary FO may inhibit endotoximia in PCOS.

### Dietary FO Restored Gut Dysbiosis in PCOS

Emerging evidences have demonstrated that the gut microbiota is an essential element in the occurrence and the development of PCOS (23, 44). In order to investigate the effect of dietary FO on the composition and the abundance levels of intestinal microbiota in PCOS, fecal samples were detected by 16S rRNA sequencing. Initially, as a result of α-diversity analysis, the abundance and the diversity of the bacterial community were assessed by observed species index and rarefaction curve. It was observed that the rarefaction curve tended to be flat when the sequence number increased to 20,000, indicating that the amount of sequencing data was reasonable (Figure S1B). The results, including both observed species index and rarefaction curve, showed no difference by Tukey test or Wilcoxon rank sum test (*P* > 0.05, Figures S1A,B).

**Figure 6:**
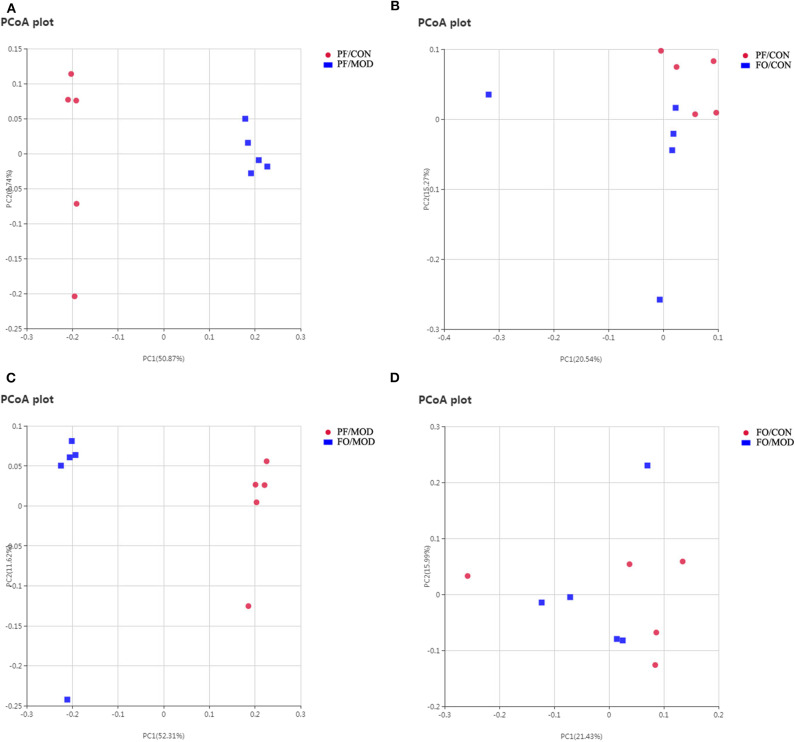
Principal coordinate analysis showing differences in fecal samples in terms of species. Beta diversity was on unweighted Unifrac. **(A)** PF/CON vs. PF/MOD. **(B)** PF/CON vs. FO/CON. **(C)** PF/MOD vs. FO/MOD. **(D)** FO/CON vs. FO/MOD.

Next, we assessed the β diversity of gut microbiota in diverse groups using unweighted principal coordinate analysis (PCoA) and weighted distance matrices (nonmetric multidimensional scaling, NMDS) (Figure 6 and Figure S2). The PCoA analysis showed that the general composition of the gut microbiota in the PF/MOD group was obviously different from that in the PF/CON or FO/MOD groups, respectively (Figure 6). Similar results were obtained from the NMDS analysis (Figure S2).

**Figure 7:**
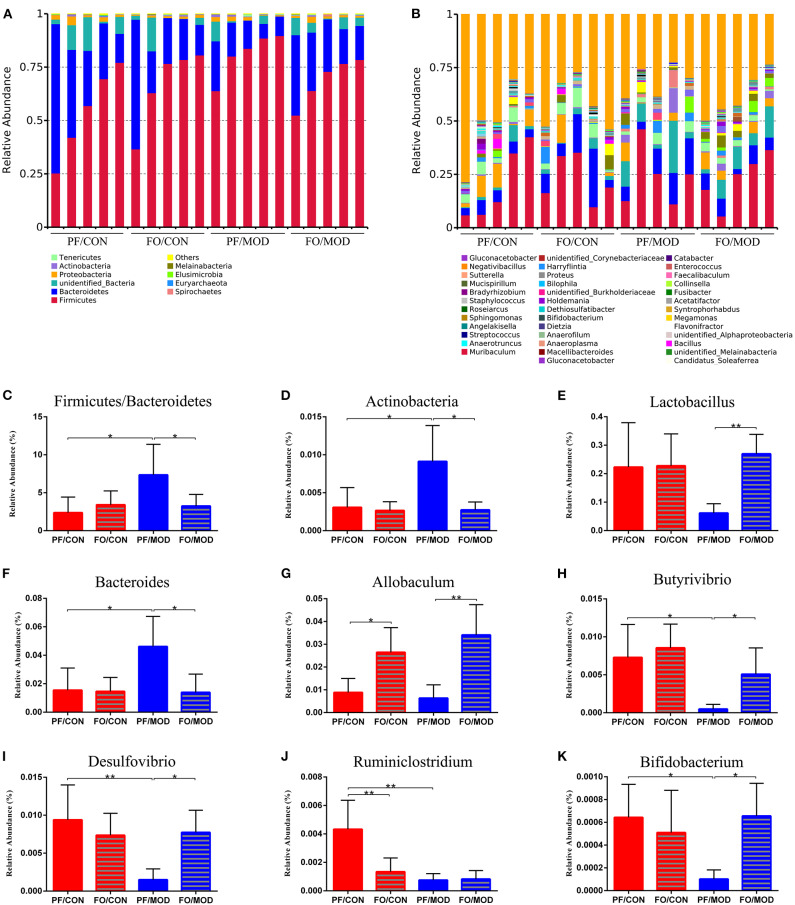
Relative abundance of gut microbial species at the phylum and the genus levels in the feces of rats. **(A)** The relative abundance of gut microbial species at the phylum level. **(B)** The relative abundance of microbial species at the genus level. **(C)** *Firmicutes*/*Bacteroidetes*. **(D)** *Actinobacteria*. **(E)** *Lactobacillus*. **(F)** *Bacteroides*. **(G)** *Allobaculum*. **(H)** *Butyrivibrio*. **(I)** *Desulfovibrio*. **(J)** *Ruminiclostridium*. **(K)** *Bifidobacterium*. Data were expressed as mean ± SD. *P < 0.05, **P < 0.01.

Finally, we investigated the difference in gut microorganism at the phylum and the genus levels in diverse groups. At the phylum level, ~90% of the gut bacteria in the diverse groups belong to *Firmicutes* and *Bacteroidetes*, another 8% comprised *Proteobacteria* and *Actinobacteria*, and the rest accounted for a low abundance (Figure 7A). The predominant *Firmicutes* and *Bacteroidetes* showed no significant change among the diverse groups. The ratio of *Firmicutes* to *Bacteroidetes* (F/B) in the PF/MOD group was higher than that in the PF/CON group (*P* = 0.0389, Figure 7C), but dietary FO decreased the F/B ratio in PCOS (*P* = 0.0453, Figure 6C). Similarly, the proportion of *Actinobacteria* in the PF/MOD group was obviously increased in comparison with that in the PF/CON group (*P* = 0.0363, Figure 7D). Dietary FO reduced the abnormal abundance of *Actinobacteria* in PCOS (*P* = 0.0182, Figure 7D). Collectively, the above results demonstrated that dietary FO supplementation had major effects on the ratio of F/B and *Actinobacteria*.

At the genus level, the relative abundance of *Lactobacillus* and *Allobaculum* in the FO/MOD group was higher than that in the PF/MOD group (*P* = 0.003, *P* = 0.0027, Figures 7E,G), whereas *Lactobacillus* and *Allobaculum* in the PF/CON group and the PF/MOD group showed no difference (*P* **>** 0.05, Figures 7E,G). The relative abundance of *Bacteroides* in the PF/MOD group was elevated compared to that in the PF/CON group (*P* = 0.0307, Figure 7F), whereas dietary FO reduced the levels of *Bacteroides* in the disease (*P* = 0.0192, Figure 7F). Moreover, the proportions of *Butyrivibrio, Desulfovibrio, Ruminiclostridium*, and *Bifidobacterium* in the PF/MOD group were obviously decreased in comparison with those in the PF/CON group (*P* = 0.0186, *P* = 0.0063, *P* = 0.0051, *P* = 0.038, Figures 7H–K). Intriguingly, dietary FO administration increased the relative abundance of *Butyrivibrio, Desulfovibrio*, and *Bifidobacterium*, compared with that in the FO/MOD group (*P* = 0.0195, *P* = 0.0026, *P* = 0.032, Figures 7H,I,K). However, dietary FO reduced the relative abundance of *Ruminiclostridium* in comparison with that in the PF/CON group (*P* = 0.0182, Figure 7J). Overall, dietary FO dramatically changed the abnormal proportions of genus components in PCOS by increasing *Lactobacillus, Allobaculum, Butyrivibrio, Desulfovibrio*, and *Bifidobacterium* as well as decreasing *Bacteroides*.

### Dietary FO Ameliorated VMB in PCOS

**Figure 8:**
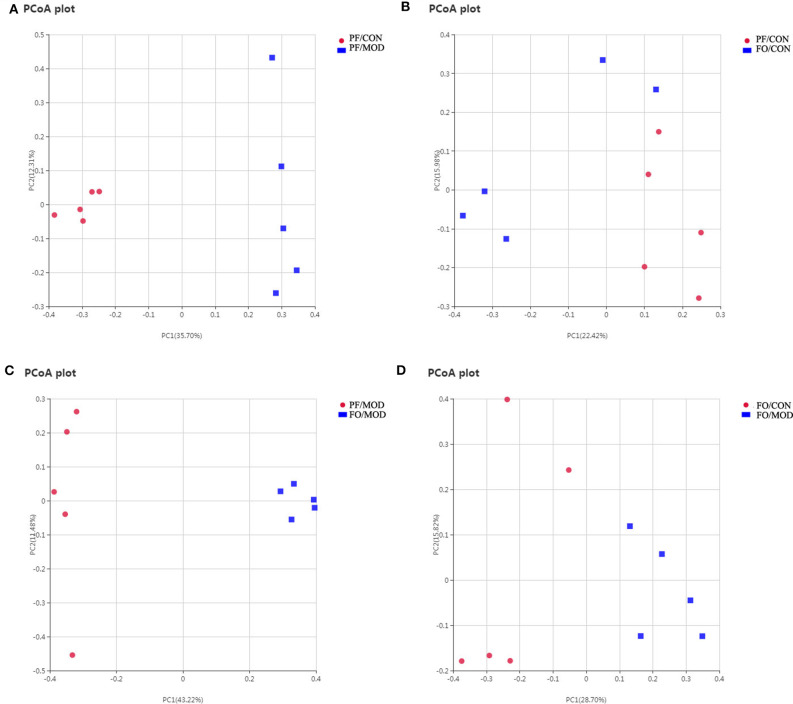
Principal coordinate analysis showing differences in vaginal secretion samples in terms of species. Beta diversity was on unweighted Unifrac. **(A)** PF/CON vs. PF/MOD. **(B)** PF/CONvs. FO/CON. **(C)** PF/MOD vs. FO/MOD. **(D)** FO/CON vs. FO/MOD.

Growing evidences have demonstrated that VMB are intimately linked to female fertility (19, 45). In order to investigate the role of VMB dysbiosis in PCOS, vaginal secretions in the four groups were detected by 16S rRNA sequencing. Observed species index showed that the abundance and the diversity of VMB in the PF/MOD group were lower than those in the PF/CON group (*P* = 0.0123, Figure S3A) and the FO/MOD group (*P* = 0.0094, Figure S3A). Moreover, rarefaction curve indicated that the amount of sequencing data was reasonable (Figure S3B). PCoA showed that VMB in the PF/MOD group was obviously different from that in the PF/CON group in terms of species in the vaginal secretions samples (Figure 8A). There was no obvious change in PCoA between the PF/CON group and the FO/CON group as well as the FO/CON group and the FO/MOD group (Figures 8B,D). Interestingly, supplementary FO seemingly altered the vaginal species compared to PF/MOD (Figure 8C). Similar results from the NMDS analysis were obtained (Figure S4).

**Figure 9:**
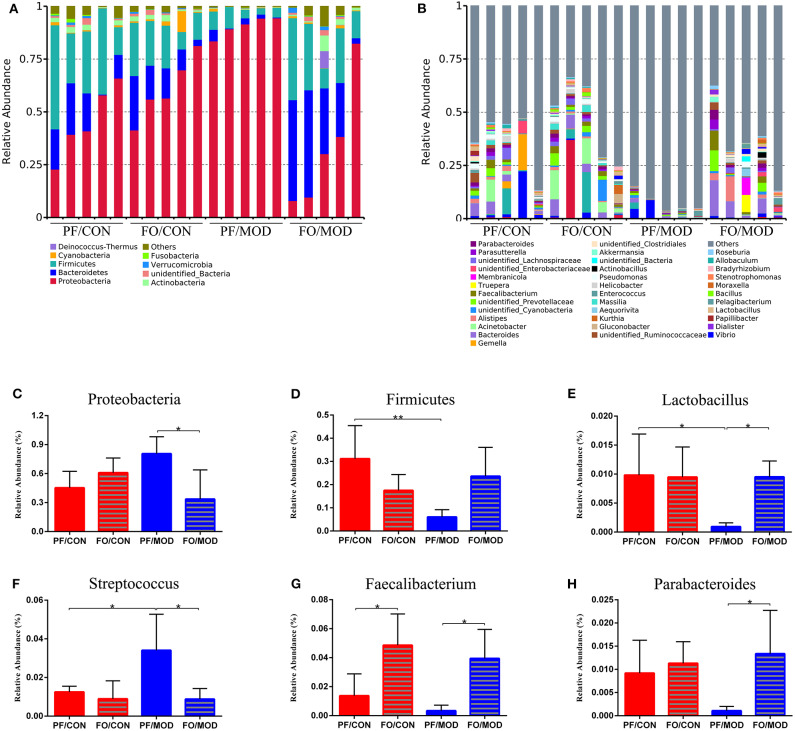
Relative abundance of vaginal microbial species at the phylum and the genus levels in the vaginal secretions of rats. **(A)** The relative abundance of vaginal microbial species at the phylum level. **(B)** The relative abundance of vaginal microbial species at the genus level. **(C)** *Proteobacteria*. **(D)** *Firmicutes*. **(E)** *Lactobacillus*. **(F)** *Streptococcus*. **(G)** *Faecalibacterium*. **(H)** *Parabacteroides*. Data were expressed as mean ± SD. *P < 0.05, **P < 0.01.

Next, we investigated the difference of VMB in the phylum and the genus levels in the diverse groups. At the phylum level, we found that *Proteobacteria, Bacteroidetes*, and *Firmicutes* were predominant in the diverse groups (Figure 9A). There was no notable difference in the relative abundances of *Proteobacteria* between the PF/CON group and the PF/MOD group (Figure 9C), whereas dietary FO decreased the proportion of *Proteobacteria* in the disease (*P* = 0.017, Figure 9C). Besides that, the level of *Firmicutes* in the PF/MOD group was lower than that in the PF/CON group (*P* = 0.0048, Figure 9D). Collectively, the above data indicated that dietary FO possessed major effects on *Proteobacteria*.

At the genus level (Figure 9B), the relative abundances of *Lactobacillus, Faecalibacterium*, and *Parabacteroides* were increased, while *Streptococcus* was decreased in the FO/MOD group compared to the PF/MOD group (*P* = 0.0101, *P* = 0.0198, *P* = 0.0188, *P* = 0.0196, Figures 9E–H). Moreover, *Lactobacilus* in the PF/MOD group was significantly decreased compared to that in the control group (*P* = 0.0234, Figure 9F). Additionally, *Streptococcus* abundance in the PF/MOD group was higher than that in the PF/CON group (*P* = 0.0339, Figure 9G). However, both *Faecalibacterium* and *Parabacteroides* in the PF/CON group and the PF/MOD group showed no difference (*P* > 0.05, Figures 9F,H). Taken together, dietary FO visibly changed the initial proportion of VMB in PCOS by increasing *Lactobacillus, Faecalibacterium*, and *Parabacteroides* as well as decreasing *Streptococcus*.

### Dietary FO Modulated the SCFAs of Gut Microbiota in PCOS

**Figure 10:**
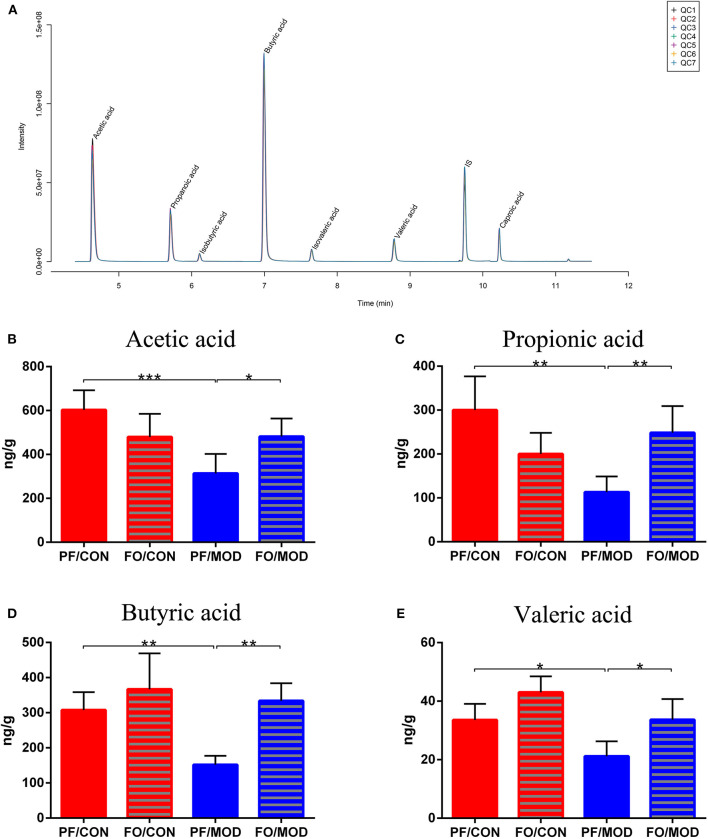
Effects of flaxseed oil on short-chain fatty acids of diverse groups. **(A)** Sample chromatogram of rats stool. **(B)** Acetic acid. **(C)** Propionic acid. **(D)** Butyric acid. **(E)** Valeric acid. Data were expressed as mean ± SD. *P < 0.05, **P < 0.01, ***P < 0.001.

SCFAs belong to the crucial metabolites of the gut microbiota, mainly including acetic acid, propionic acid, butyric acid, and pentanoic acid. The TIC chromatogram showed that every SCFA can be distinguished clearly and exhibited a good peak shape, indicating that the method and data were reliable (Figure 10A). As a result of detection by liquid chromatography-mass spectrometry, acetic acid (*P* = 0.0009), propionic acid (*P* = 0.0011), butyric acid (*P* = 0.003) as well as pentanoic acid (*P* = 0.0160) in the PF/MOD group were significantly decreased compared with those in the PF/CON group (Figures 10B–E). Intriguingly, the decreased levels of SCFAs in PCOS were restored by dietary FO administration (acetic acid: *P* = 0.0145, propionic acid: *P* = 0.0027, butyric acid: *P* = 0.001, and pentanoic acid: *P* = 0.0124, Figures 10B–E).

### Correlation Analysis

**Figure 11:**
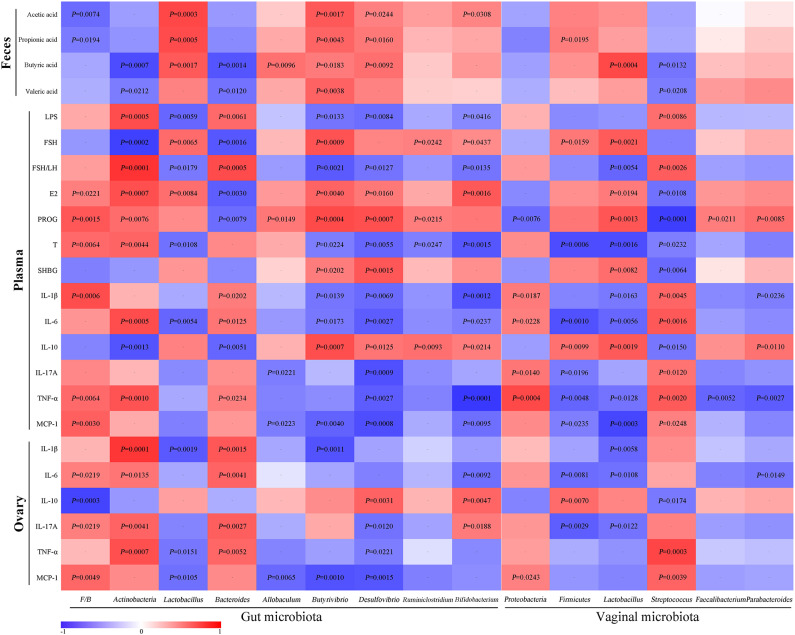
Correlation analyses between relative abundance (%) of microbiota and other related indicators.

For the assessment of relationships among hormones, gut microbiota/VMB, and inflammation in PCOS, we performed a correlation analysis (Figure 11). The abundance of anti-inflammatory bacteria including *Lactobacillus, Firmicutes, Butyrivibrio, Desulfovibrio*, and *Bifidobacterium* were positively correlated with SCFAs, FSH, E2, PROG, and IL-10, respectively. However, they were negatively associated with LPS, FSH/LH, T, and pro-inflammatory indicators. Reversely, the abundance of “bad bacteria” including *Actinobacteria, Bacteroides*, and *Streptococcus* as well as the F/B ratio showed a negative correlation with SCFAs, FSH, E2, PROG, and IL-10, respectively. Moreover, the abovementioned pro-inflammatory bacteria were positively correlated with LPS, FSH/LH, T, and pro-inflammatory indicators, respectively. Additionally, the rest of the different bacteria, including *Allobaculum, Ruminiclostridium, Proteobacteria, Faecalibacterium*, and *Parabacteroides*, were only connected with a few parameters, such as PROG. Taken together, there were close interactions and correlations among gut/vaginal bacteria, inflammation, and sex steroid hormones.

## Discussion

In the present study, the therapeutic effects and the associated mechanisms of dietary FO supplementation on letrozole-induced PCOS rats were investigated through determining the sex steroid hormones, lipid metabolism, insulin resistance, inflammation, and gut/vaginal microbiota. Here we demonstrated that the potent effects of dietary FO on PCOS may integrally be due to modulating the hormones–inflammation–gut/vaginal microbiota axis, potentially providing an inexpensive intervention for the control of PCOS.

Firstly, the letrozole-induced PCOS model used in this study has been widely utilized to assess the effects and the mechanisms of interventions on precautions and pathogenesis in PCOS (46–48). Letrozole, an aromatase inhibitor, induces hyperandrogenemia by reducing the conversion of T to estrogen (49). Accumulated human and animal studies have demonstrated that omega-3 PUFAs docosahexenoic acid (DHA) and eicosapntemacnioc acid (EPA) may ameliorate PCOS progression (50, 51). Anovulation was dramatically improved by DHA supplementation (52). In this study, the improvement of the disrupted estrous cycle and ovulatory function after dietary FO treatment demonstrated that ALA-rich FO possessed the ability to ameliorate PCOS, indicating that inexpensive FO exhibited similar effects with EPA and DHA. We speculate that *in vivo* interconversion among FO, EPA, and DHA may partially contribute to understanding this effect of omega-3 PUFAs. However, the dynamic interconversion after FO supplementation needs to be further determined.

PCOS represents a chronic endocrine–metabolic disease with hyperandrogen and low estrogen (53). The LH/FSH ratio was regarded as a main biomarker of diagnosis in PCOS (54, 55). The notably increased T level is usually considered as a marker of hyperandrogenism in PCOS (56). During the development and the progression of PCOS, abnormally increased T secretion suppresses the production of E2, SHBG, and FSH (57–59). Furthermore, the suppressed FSH results in the arrest of ovarian folliculogenesis and corpora lutea under the synergistic effect of LH, thereby decreasing the PROG produced by the corpus luteum (60). SHBG produced by the liver, a transporter of sex hormones with a high affinity for T but a low affinity for E2, can be used to evaluate the severity of hyperandrogenism and the therapeutic efficacy (61). In this study, abnormal elevated plasma T and LH/FSH ratio, as well as reduced levels of plasma FSH, E2, SHBG, and PROG in PCOS, was notably rectified by FO intervention, demonstrating that dietary FO supplementation was capable of improving the homeostasis of sex steroid hormones in PCOS. A study indicates that an intake of dietary EPA exerted beneficial effects on androgens in PCOS (62). Moreover, EPA treatment increases the number of follicular cell layers and decreased the levels of LH and T in PCOS (27).

Recent studies have shown that patients with PCOS were mainly accompanied with dyslipidemia and insulin resistance, which might be caused by hyperandrogenism (63, 64). Omega-3 fatty acids may be recommended for the treatment of PCOS patients with insulin resistance as well as high TC and TG (51). Consistent with previous findings (65, 66), decreases of TC, TG, and LDL levels, but an increase of HDL, after dietary FO treatment demonstrated that dietary FO improved dyslipidemia in PCOS. Additionally, we found that dietary FO intervention reduced the levels of FINS and HOMA-IR, which was consistent with the findings of previous studies (67, 68). Supplementation of fish oil rich in omega-3 fatty acids can elevate the adiponectin levels to stimulate AMPK in the muscle to downstream oxidative pathways and to finally improve insulin sensitivity (67, 68). However, the role of the AMPK pathway in the amelioration of insulin resistance after dietary FO supplementation needed to be further investigated.

Numerous studies have demonstrated that a chronic low-grade degree of inflammation plays a critical role in the development of PCOS (69, 70). In our study, we indicated that dietary ALA-rich FO alleviated systematic and ovarian inflammation *via* suppressing the pro-inflammatory cytokines (TNF-α, IL-1β, IL-6, IL-17A, and MCP-1) and elevating the anti-inflammatory IL-10, suggesting the anti-inflammation role of inexpensive dietary FO administration in PCOS. A previous report illustrated that omega-3 PUFAs and vitamin E co-supplementation down-regulated IL-8 and TNF-α expression in PCOS patients (71). Dietary ALA-rich FO inhibited the production of TNF-α, IL-1β, and IL-6 in T2DM and ALD (37, 38). EPA treatment can decrease IL-1β and TNF-α but increase IL-10 in PCOS (27). Clinical studies have shown that the supplementation of omega-3 PUFAs is beneficial in decreasing the MCP-1 expression in macrophages and the levels of endothelial chemokine (72, 73). Due to accumulating evidences on the critical role of macrophages in the pathogenesis of PCOS, we speculate that the anti-inflammatory effect of dietary FO may attribute to the inhibition of the activation of macrophages and its polarization. The exact role of the macrophage in the underlying mechanism of FO-treated PCOS needs to be further investigated.

Growing evidences have demonstrated that the gut microbiota and their metabolites are closely associated with the occurrence and the development of PCOS (18, 74–76). Liu et al. found a decrease in *Ruminococcaceae* and an increase in gram-negative bacteria, including *Bacteroides* and *Desulfovibrio*, in women with PCOS, which was consistent with our research (20). Regarding to the phylum level of the gut microbiota, we found that the abundances of *Firmicutes, Bacteroidetes*, and *Proteobacteria* were the most dominant in all groups, in consistence with previous studies (12, 77). An increase in *Firmicutes*/*Bacteroidetes* ratio is closely related to obesity (12, 78). We found that the increased ratio of F/B in PCOS was rectified by dietary FO administration. Moreover, in the genus level, our results showed that FO restored gut dysbiosis in PCOS by up-regulating *Lactobacillus, Allobaculum, Desulfovibrio*, and *Bifidobacterium* and down-regulating *Actinobacteria* and *Bacteroides*, suggesting that dietary FO attenuated PCOS *via* restoring the gut dysbiosis. Similarly, dietary prebiotic inulin supplementation ameliorated PCOS in mice *via* improving the gut microbiota (23). A recent clinical study found that *Bifidobacterium lactis* V9 was significantly decreased in patients with PCOS. This probiotic regulated the secretion of sex hormones in PCOS patients through the gut–brain axis (22).

Microbial LPS and SCFAs are thought to play a pivotal role in regulating inflammation and metabolism in PCOS (79, 80). LPS, derived from gram-negative bacteria, activates the inflammation of the peripheral circulation and the ovary through the TLR4-NF-κB signaling pathway in macrophage to release inflammatory cytokines (74). In this study, abnormal plasma LPS was restored by dietary FO treatment, demonstrating that FO improved gut dysbiosis and the gut barrier to decrease the intestinal permeability of LPS translocation from the intestines to the systematic circulation in PCOS, finally contributing to the suppression of systematic and ovarian inflammation. In addition, the microbial fermentation end-products SCFAs are pivotal in the regulation of intestinal permeability and inflammation in the gut and the systemic circulation (81). The anti-inflammatory and the immunomodulatory effects of SCFAs might be due to the activation of specific cell receptors G-protein-coupled receptor (GPR) 109a, GPR41, and GPR43 and the intracellular target *via* inhibiting histone deacetylase activity (82–85). In this study, elevated SCFA levels, including acetic acid, propionic acid, butyric acid, and valeric acid, after dietary FO treatment may subsequently result in enhancing the integrity of the intestinal mucosal barrier as well as inhibiting enteral and parenteral inflammation. We consider that the alteration of SCFAs may probably regulate inflammation through the SCFAs–GPR–inflammatory cells signaling pathway, which is ongoing to investigated in our subsequent research. Additionally, other microbial metabolites may be involved in the effects of dietary FO intervention on PCOS and need to be further investigated using metabonomics methodology.

Studies have indicated that VMB plays a crucial role in the development of reproductive and HPV/HIV infectious diseases (86–89). However, the effects of the proportions of VMB on PCOS are rarely reported. In this study, we firstly demonstrated a reduction of the abundance and the diversity of VMB in PCOS. The richness and the diversity of the VMB significantly affected host reproductive ability, metabolic function, as well as the defense ability of immune system (90). A reduction in VMB diversity led to a lack of estrogen-metabolizing bacteria and thereby decreasing the circulating estrogen, which may induce a hypoestrogen-related disease. Moreover, we found that dietary FO altered VMB *via* up-regulating *Lactobacillius, Faecalibacterium*, and *Parabacteroides* as well as down-regulating *Proteobacteria* and *Streptococcus*. *Lactobacillus* in VMB can generate acidic fermentation products (primarily lactic acid) which serve to create an acidic environment to restrict the growth of most pathogens in the vagina (91). The ability of *Lactobacillus* to inhibit infection without inducing inflammation may maximize fecundity and successful pregnancy outcome in women (92, 93). Additionally, *Faecalibacterium* is a commensal bacterium, the absence of which may be associated with lipid metabolism and inflammation in Crohn's disease (94). Studies on the effects of VMB in reproductive and gynecological diseases are still limited and largely unknown, which needs to be further studied.

In this study, we found that sex steroid hormones, gut/vaginal microbiota, and inflammation were closely correlated. The abundances of beneficial bacteria (*Lactobacillus, Firmicutes, Butyrivibrio, Desulfovibrio*, and *Bifidobacterium*) were positively correlated with SCFAs and E2, while these are negatively associated with LPS, T, and pro-inflammatory indicators. Reversely, *Actinobacteria, Bacteroides*, and *Streptococcus* were negatively correlated with SCFAs and E2, whereas these were positively correlated with LPS, T, and pro-inflammatory indicators. Recent studies indicated the bi-direction regulation of the gut microbiota and circulating estrogen levels (95). E2 level may improve bacterial virulence by inhibiting quorum sensing signaling. PROG has been proven to promote the growth of *Bacteroides* and *Prevotella* (96, 97). The gut microbiome promotes the enterohepatic circulation of estrogenic metabolites in the host through the secretion of β-glucuronidase enzymes to hydrolyze estrogenic glucuronides, increasing the reabsorption of estrogens into the blood and reducing elimination from the body (95, 98, 99). Additionally, the microbial fermentation end-products SCFAs (butyrate) can suppress the levels of pro-inflammation cytokines and LPS and enhance the gut mucosa integrity by up-regulating the expression of tight junction proteins and the production of retinoic acid (100–102).

## Conclusions

This study highlighted that dietary FO ameliorated PCOS *via* the sex steroid hormones–gut/vaginal microbiota–inflammation axis in rats, which may potentially serve as an inexpensive intervention for the control of PCOS patients, especially those who are vegetarians.

## Data Availability Statement

The datasets presented in this study can be found in online repositories. The names of the repository/repositories and accession number(s) can be found below: NCBI, accession number PRJNA624034.

## Ethics Statement

The animal study was reviewed and approved by the Ethics Committee of Ningxia Medical University (No. 2016-017).

## Author Contributions

HW, TW, and XZ designed and wrote the paper. TW, LS, YL, LZ, ZW, KL, HL, TB, LG, HW, and XZ performed the research. All authors have read and approved the final manuscript.

## Conflict of Interest

The authors declare that the research was conducted in the absence of any commercial or financial relationships that could be construed as a potential conflict of interest.

## Supplementary Material

Figure S1 Alpha-diversity analysis showing differences in diverse groups in terms of abundance and diversity of gut microbiota. **(A)** Observed species index. **(B)** Rarefaction curve.

Figure S2 Nonmetric multidimensional scaling analysis showing differences in fecal samples in terms of species. Beta diversity was on weighted distance matrices. **(A)** PF/CON *vs*. PF/MOD. **(B)** PF/CON *vs*. FO/CON. **(C)** PF/MOD vs. FO/MOD. **(D)** FO/CON vs. FO/MOD.

Figure S3 Alpha-diversity analysis showing differences in diverse groups in terms of abundance and diversity of vaginal microbiota. **(A)** Observed species index. **(B)** Rarefaction curve.

Figure S4 Nonmetric multidimensional scaling analysis showing differences in vaginal secretion samples in terms of species. Beta diversity was on weighted distance matrices. **(A)** PF/CON *vs*. PF/MOD. **(B)** PF/CON vs. FO/CON. **(C)** PF/MOD vs. FO/MOD. **(D)** FO/CON vs. FO/MOD.

Table S1 Determination of fatty acids in organic flaxseed oil.
