# Supplementary figures and images for: Dietary α-Linolenic Acid-Rich Flaxseed Oil Exerts Beneficial Effects on Polycystic Ovary Syndrome Through Sex Steroid Hormones—Microbiota—Inflammation Axis in Rats
Source: Front Endocrinol (Lausanne). 2020 May 27;11:284. doi: 10.3389/fendo.2020.00284 (PMC7326049; doi:10.3389/fendo.2020.00284)

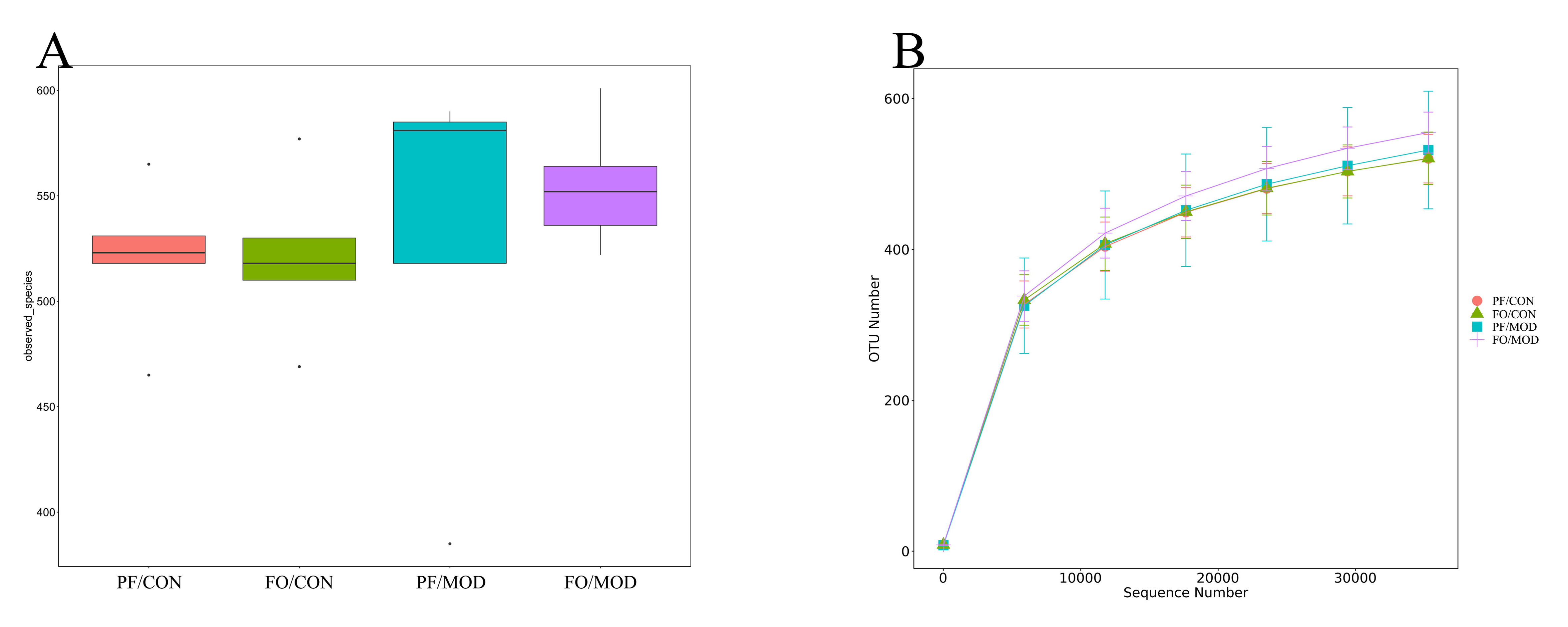

Supplement: Figure S1 — Alpha-diversity analysis showing differences in diverse groups in terms of abundance and diversity of gut microbiota. (A) Observed species index. (B) Rarefaction curve. [file Image_1.JPEG]

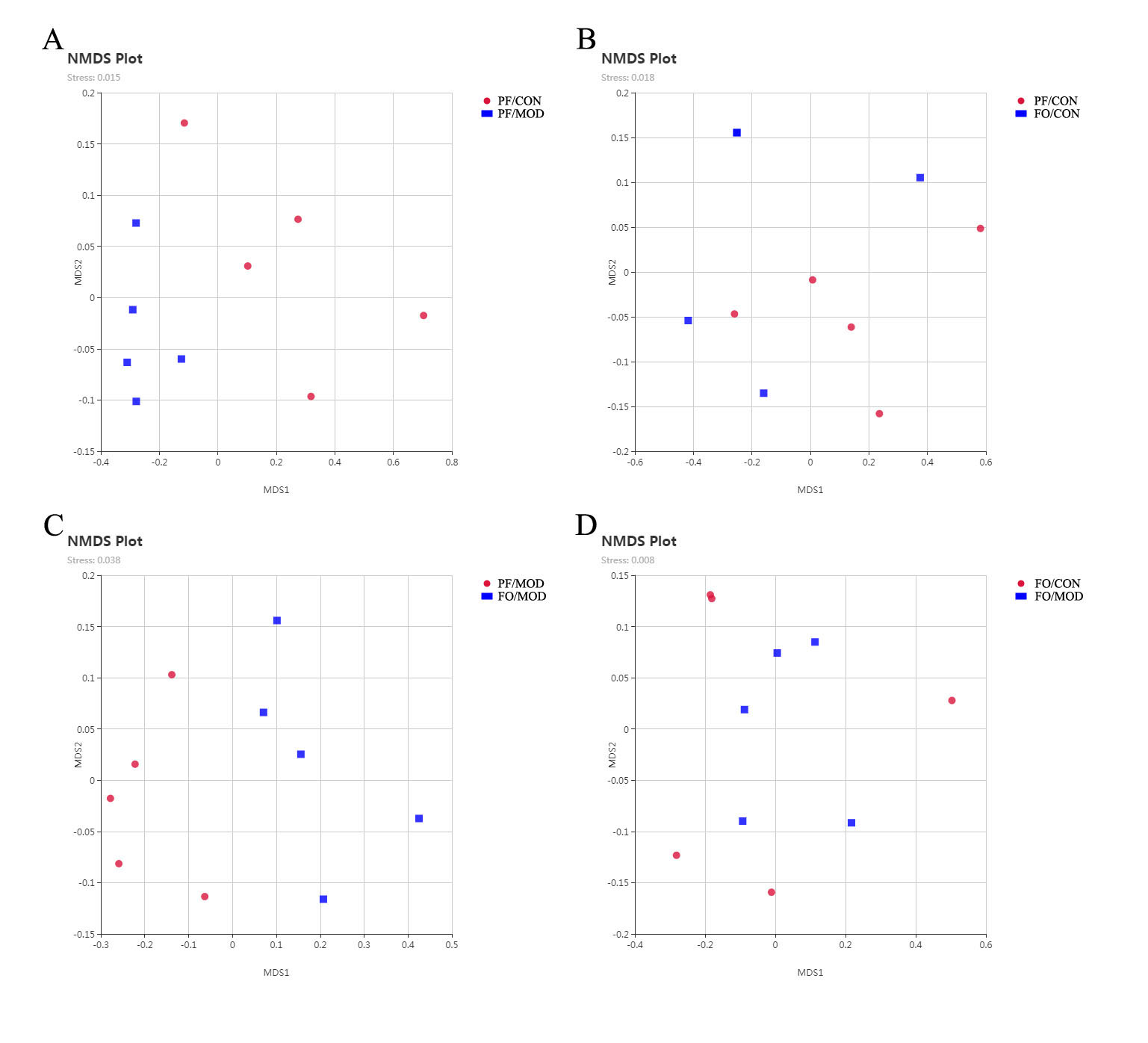

Supplement: Figure S2 — Nonmetric multidimensional scaling analysis showing differences in fecal samples in terms of species. Beta diversity was on weighted distance matrices. (A) PF/CON vs. PF/MOD. (B) PF/CON vs. FO/CON. (C) PF/MOD vs. FO/MOD. (D) FO/CON vs. FO/MOD. [file Image_2.JPEG]

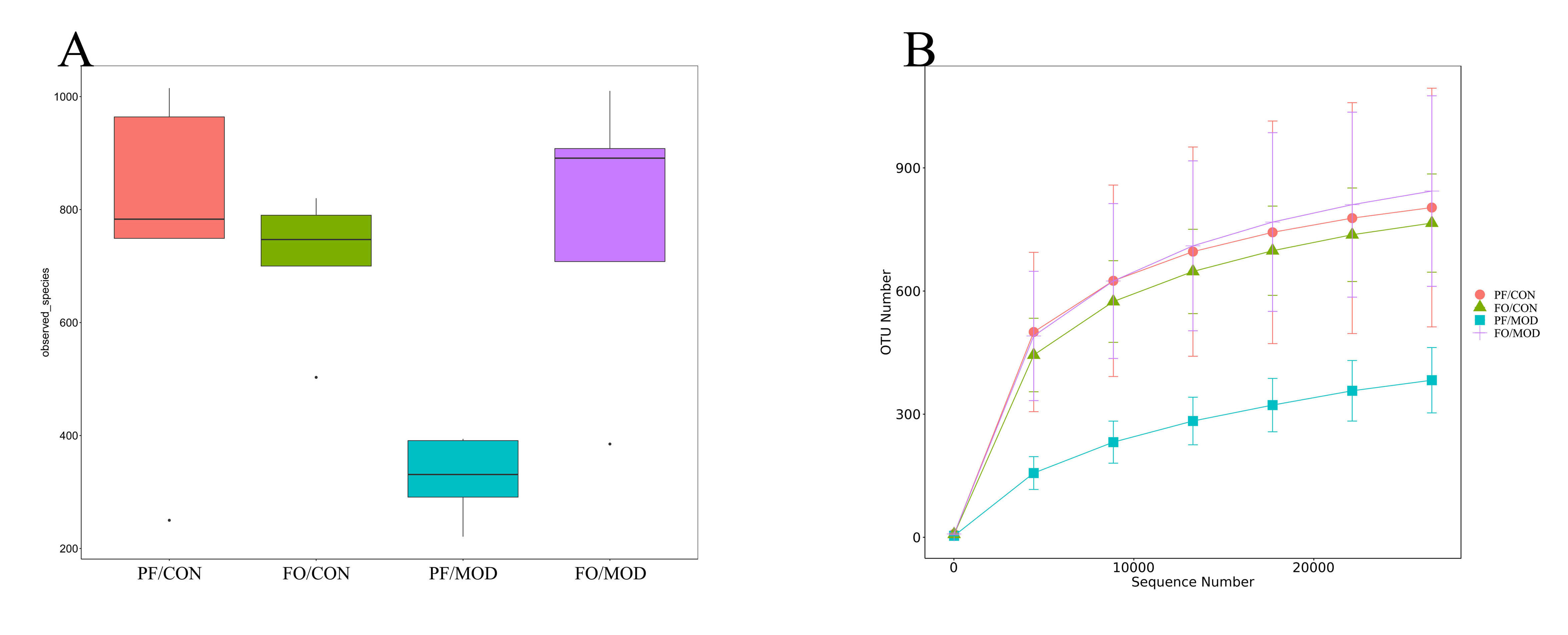

Supplement: Figure S3 — Alpha-diversity analysis showing differences in diverse groups in terms of abundance and diversity of vaginal microbiota. (A) Observed species index. (B) Rarefaction curve. [file Image_3.JPEG]

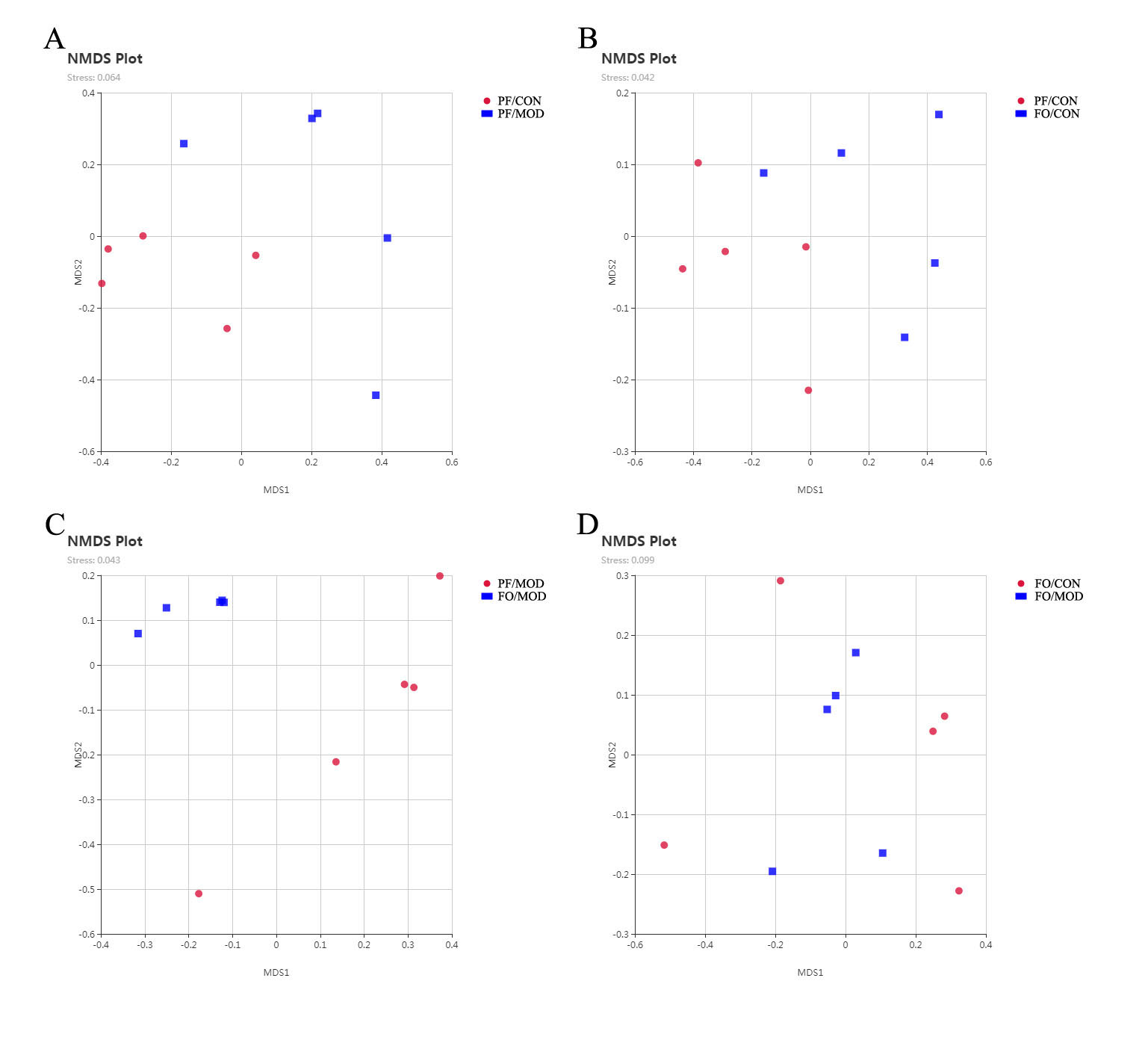

Supplement: Figure S4 — Nonmetric multidimensional scaling analysis showing differences in vaginal secretion samples in terms of species. Beta diversity was on weighted distance matrices. (A) PF/CON vs. PF/MOD. (B) PF/CON vs. FO/CON. (C) PF/MOD vs. FO/MOD. (D) FO/CON vs. FO/MOD. [file Image_4.JPEG]
