# Supplementary material for: Dietary α-Linolenic Acid-Rich Flaxseed Oil Exerts Beneficial Effects on Polycystic Ovary Syndrome Through Sex Steroid Hormones—Microbiota—Inflammation Axis in Rats
Source: Front Endocrinol (Lausanne). 2020 May 27;11:284. doi: 10.3389/fendo.2020.00284 (PMC7326049; doi:10.3389/fendo.2020.00284)
Supplement: Table S1 — Determination of fatty acids in organic flaxseed oil. [file Table_1.DOC]

**Table 1** Determination of fatty acids in organic flaxseed oil.

| **Fatty acid** | | **%** |
| --- | --- | --- |
| **[saturated](../../../../C:/Program%20Files%20(x86)/Youdao/Dict/8.6.2.0/resultui/html/index.html#/javascript:;) [fatty](../../../../C:/Program%20Files%20(x86)/Youdao/Dict/8.6.2.0/resultui/html/index.html#/javascript:;) [acid](../../../../C:/Program%20Files%20(x86)/Youdao/Dict/8.6.2.0/resultui/html/index.html#/javascript:;)** |  | **9.6** |
| Lauric acid | C12:0 | 0.0036 |
| Myristic acid | C14:0 | 0.0374 |
| Pentadecanoic acid | C15:0 | 0.0162 |
| Cetylic acid | C16:0 | 5.43 |
| Heptadecanoic acid | C17:0 | 0.049 |
| Stearic acid | C18:0 | 3.76 |
| Arachidic acid | C20:0 | 0.118 |
| Behenic acid | C22:0 | 0.111 |
| 23-carbonic acid | C23:0 | 0.0101 |
| Tetracosanoic acid | C24:0 | 0.0687 |
|  |  |  |
| **unsaturated fatty acid** |  | **90.47** |
| Palm monoenic acid | C16:1n9 | 0.0579 |
| Oleic acid | C18:1n9 | 18.1 |
| Linoleic acid | C18:2n6 | 16.1 |
| Arachidonic acid | C20:1 | 0.394 |
| Cis-, cis-11, 14-eicosenoic acid | C20:2 | 0.0205 |
| Cis-13-docosenoic acid | C22:1n9 | 0.0315 |
| Cis-11,14,17-eicosenoic acid | C20:3n3 | 0.0606 |
| arachidonic acid | C20:4n6 | 0.0086 |
| Linolenic acid | C18:3n6c + C18:3n3 | 55.7 |
|  |  |  |
| ω-3 ƩPUFA |  | 55.0 |
| ω-6 ƩPUFA |  | 16.8 |
| ω-3/ω-6 |  | 3.27 |
| ω-3+ω-6 |  | 71.8 |
| ω-9 ƩPUFA |  | 18.5 |
